# Effects of Neohesperidin Dihydrochalcone (NHDC) on Oxidative Phosphorylation, Cytokine Production, and Lipid Deposition

**DOI:** 10.3390/foods10061408

**Published:** 2021-06-18

**Authors:** Sooyeon Choi, Seungmin Yu, Jonghun Lee, Wooki Kim

**Affiliations:** 1 Department of Food Science and Biotechnology, Kyung Hee University, Yongin 17104, Korea; kiss_me94@naver.com (S.C.); dbtmdals1004@khu.ac.kr (S.Y.); 2 Department of Food Science and Biotechnology, Gachon University, Seongnam 13120, Korea; foodguy@gachon.ac.kr

**Keywords:** NHDC, chalcone, inflammation, obesity, lipid

## Abstract

The sweetener neohesperidin dihydrochalcone (NHDC) is a precursor for anthocyanins and has been reported to have various bioactivities, including antioxidant and hepatitis inhibitory effects. However, its inflammatory functions and mechanisms of action are poorly understood. In this study, RAW 264.7 murine macrophages were treated with NHDC and its metabolite dihydrocaffeic acid (DHCA), after which cytokine production and mitochondrial respiration were assessed. DHCA significantly down-regulated the secretion of pro-inflammatory cytokines. In contrast, NHDC had a marginal effect, suggesting that the biological metabolism of NHDC to DHCA is required for its anti-inflammatory function. However, both NHDC and DHCA rescued LPS-induced suppression of oxidative phosphorylation, which is a hallmark of anti-inflammatory M2 macrophages. 3T3-L1 adipocytes showed lower fat deposition in the presence of DHCA, while sugar-containing NHDC showed a slight increase in fat deposition. In high-fat diet-induced obese mice, treatment with NHDC successfully down-regulated body weight gain in a dose-dependent manner. Furthermore, M2 polarized bone-marrow-derived macrophages (BMDM) from NHDC-fed mice secreted an increased amount of the anti-inflammatory cytokine IL-10. Overall, these results indicate that NHDC and its physiological metabolite DHCA have the potential to suppress the inflammatory response and obese status.

## 1. Introduction

Inflammation is a complex immune reaction involving innate and adaptive cells, blood vessels and molecular mediators to remove non-self materials and promote wound healing [1], characterized by fever, swelling and redness [2]. Macrophages play a pivotal role in these inflammatory responses given their phagocytic action and production of cytokines and chemokines [3]. In addition, macrophages also participate in inflammatory responses by expressing proteins for antigen presentation and co-stimulation to adaptive immune cells [4]. Inflammatory macrophages tend to exhibit reduced mitochondrial respiration, which can be quantified by down-regulated oxygen consumption in the micromilieu of the cellular environment [5]. The aforementioned macrophages in inflammatory status are called activated M1 macrophages. The experimental polarization to M1 cells was established using lipopolysaccharides (LPS) or interferon gamma (INF-γ). In contrast, alternative macrophage activation was discovered later and involves M2 macrophages. These M2 cells, which can be experimentally polarized by treatment with interleukin (IL)-4 in a culture medium, are unique in their anti-inflammatory and tissue repairing functions, in part, by the secretion of anti-inflammatory cytokine IL-10 [6]. Moreover, M2 macrophages are known to shift cellular energy metabolism from glycolysis to oxidative phosphorylation, resulting in increased oxygen consumption. There have been several approaches to regulate inflammatory macrophage functioning by reprogramming mitochondrial respiration using dietary ingredients [7,8,9].

Obesity is associated with various metabolic syndromes, including insulin resistance and type 2 diabetes [10]. Obesity also creates a chronic inflammatory state through the direct accumulation of M1 macrophages in obese tissues and through the indirect activation of immune cells by adipocyte-produced cytokines and adipokines [11]. Several obesity-induced inflammation models in mice have been studied [12,13], suggesting that blood lipid metabolic markers, both in vitro and in vivo, are useful in identifying inflammatory states.

Neohesperidin dihydrochalcone (NHDC, CAS No. 20702-77-6, Sigma-Aldrich, St. Louis, MO, USA) is commercially synthesized by the catalytic alkali hydrogenation of hesperidin, a flavonoid that is extracted from citrus peels. Even though there is no evidence that NHDC is naturally produced, several studies have described biotransformation pathways which, in part, were shared by anthocyanin production [14]. As a food ingredient, it is noteworthy that NHDC has 250–1800 times the sweetness of sucrose [15], even though direct replacement of sucrose with artificial sweeteners is limited to beverages and not feasible in general due to physical and/or chemical properties, including bulk, texture, and flavor formation [16]. Prior studies have found that NHDC has no toxicity on cells and animals [17,18], while its antioxidative, anti-inflammatory and anti-apoptotic properties were reported [19,20]. In fact, naturally produced chalcones and hesperidin were also shown to have these beneficial effects [21,22,23]. Therefore, it is reasonable to hypothesize that the health-beneficial activities of these molecules share an advantageous structure. After oral consumption, NHDC is degraded into 3-(3,4-dihydroxyphenyl)propionic acid, also named dihydrocaffeic acid (DHCA; CAS No. 1078-61-1, Sigma-Aldrich, St. Louis, MO, USA), by intestinal microbiota [24]. The current study evaluated the anti-inflammatory mechanisms of NHDC, as well as its metabolite DHCA, in macrophages and adipocytes. We also performed a dietary study of NHDC in high-fat diet-induced obese mice.

## 2. Materials and Methods

### 2.1. Cell Culture and Treatment

The murine macrophage cell line, RAW 264.7, was purchased from the Korean Cell Line Bank (Seoul, Korea). The cells were incubated in Dulbecco’s modified Eagle’s medium (DMEM) supplemented with 10% fetal bovine serum (FBS) and 1% penicillin/streptomycin at 37 °C in 5% CO_2_. NHDC and DHCA were purchased from Sigma-Aldrich and treated to RAW 264.7 for 24 h at the designated concentration. The cells were stimulated with LPS at 500 ng/mL for 12 h, and the media was harvested for cytokine quantification.

### 2.2. Cytokine Quantification

The pro-inflammatory cytokines, IL-6 and TNF-a, in the culture medium were assessed using an enzyme-linked immunosorbent assay (ELISA)kit (BD Bioscience, Carlsbad, CA, USA) following the manufacturer’s instructions as previously reported [25]. Briefly, 96-well plates were coated with specific capture antibodies overnight at 4 °C. To prevent non-specific protein binding, the assay diluent was treated for 1 h, followed by the addition of culture supernatants. After 2 h of incubation at room temperature, the wells were washed. Next, a biotinylated detection antibody and streptavidin conjugated with horseradish peroxidase (SAv-HRP) were added for 1h. Subsequently, the substrate solution was treated to each well and incubated for 30 min in the dark. Finally, a stop solution was added, and the cytokine concentration was measured using a microplate reader (Bio-Rad, Hercules, CA, USA) at 450 nm.

### 2.3. Surface Activation Protein Measurement

Cellular surface proteins were quantified using fluorescent antibody staining followed by a flow cytometric analysis. Briefly, RAW 264.7 cells treated with NHDC or DHCA followed by LPS-stimulation were washed with PBS and incubated for 10 min at 4 °C with anti-mouse CD16/CD32 mAbs (Fc Block, eBioscience, San Diego, CA, USA) to prevent non-specific antibody binding. The cells were subsequently stained with fluorescence-conjugated mAbs; fluorescein isothiocyanate-conjugated anti-mouse CD80 (CD80-FITC), phycoerythrin-conjugated anti-mouse CD86 (CD86-PE) and allophycocyanin-conjugated anti-mouse MHC class II (MHC II-APC) at 4 °C for 10 min. The wells were washed by the addition of excess PBS and centrifugation at 300× *g* for 5 min. The cells were then resuspended in PBS and analyzed using a flow cytometer (BD Accuri C6, BD Biosciences, Carlsbad, CA, USA) for the quantification of specific protein expression on macrophage surfaces. The mean fluorescence intensities (MFIs) were determined by BD Accuri^TM^ C6 software (BD Biosciences, Carlsbad, CA, USA).

### 2.4. Cell Mitostress Test

The RAW 264.7 cells were seeded at 1 × 10^4^ cells per well in Seahorse^TM^ XFp assay miniplates (Agilent Technologies, Santa Clara, CA, USA). The cells were further treated with NHDC and DHCA for 24 h at 37 °C in a 5% CO_2_ incubator. The cells were then stimulated with LPS (500 ng/mL) for another 24 h. The cells were further cultivated with DMEM containing 4500 mg/L D-glucose, L-glutamine, and sodium pyruvate at pH 7.4. They were then incubated at 37 °C in a non-CO_2_ incubator for 1 h. The plates were mounted on the extracellular flux analyzer. Specific inhibitors of ATP synthase, oligomycin (1.0 µM), mitochondrial uncoupler, FCCP (1.0 µM), and rotenone/antimycin A (0.5 µM) were added to the media sequentially, according to the manufacturer’s instructions. The oxygen consumption rate (OCR), an indicator of oxidative phosphorylation, was automatically measured using the Seahorse^TM^ XFp analyzer and software (Agilent Technologies) to assess the OCR for basal respiration, maximal respiration and ATP production according to the following equations:

Basal respiration = OCR before oligomycin—OCR after rotenone/antimycin A(1)

Maximal respiration = Maximum OCR after FCCP—OCR after rotenone/antimycin A(2)

ATP production = OCR before oligomycin—OCR after oligomycin(3)

### 2.5. Lipid Accumulation Assessment

To assess the lipid accumulation, the oil red O (ORO) staining method was used in the 3T3-L1 cells. Pre-adipocytes were seeded on 24-well plates at 5 × 10^4^ cells/well and differentiated to adipocytes with DMEM containing 10% FBS, 10 μg/mL insulin, 1 μM dexamethasone, and 0.5 mM 3-isobutyl-1-methylxanthine for 2 days. Afterwards, the medium was changed to DMEM containing insulin (10 μg/mL) and replaced every 2 days until day 8 in the presence of either NHDC or DHCA, followed by fixation in 10% formalin at RT. The formalin was washed with 60% isopropanol, and the fixed cells were treated with ORO solution for 10 min at RT. The excess ORO solution was removed, and the cells were immediately washed with distilled water four times. The lipid accumulation was measured by the dissolved ORO solution in 100% isopropanol using a microplate reader (Bio-Rad) at 490 nm, with normalization to initial culture cell number and non-differentiated control.

### 2.6. Animal Feeding and Blood Analysis

Male C57BL/6 mice (4 weeks, 13–16 g) were purchased from Raon Bio (Yongin-si, Korea) and acclimated with a rodent normal diet (ND) for one week under a 20 °C climate-controlled 12/12 h dark/light cycle. Table 1 shows the experimental high-fat diets (HFD) with lower or higher contents of NHDC and the control diets. The animals were randomly divided into four groups (*n* = 10) and fed the respective diet and water for 11 weeks ad libitum. The body weight was measured every week. After the last body weight measurement, the mice were euthanized by CO_2_ inhalation.

### 2.7. M2 Polarization of Bone Marrow-Derived Macrophages

The bone marrow cells were separated from the tibias and femurs. These cells at 5 × 10^5^ cells/well were cultured in Iscove’s modified Dulbecco’s medium (IMDM, Thermo Fisher Scientific, Waltham, MA, USA) that was supplemented with 10% FBS and 10 ng/mL macrophage colony-stimulating factor (M-CSF, Thermo Fisher Scientific) for 7 days to induce the bone marrow-derived macrophages (BMDM). BMDM were further polarized to anti-inflammatory M2 macrophages in the presence of 100 ng/mL IL-4. Anti-inflammatory M2 cytokine IL-10 was quantified by using a specific ELISA kit (BD Biosciences) with normalization to the initial culture cell number.

### 2.8. Statistical Analysis

The data are presented as means ± standard errors of the mean (SEM). The statistical significance of differences among the groups was demonstrated by one-way ANOVA, followed by Tukey’s post-hoc multiple comparisons. GraphPad Prism 5 software (La Jolla, CA, USA) was used. The *p* values < 0.05 were considered significant.

## 3. Results and Discussion

### 3.1. Suppressed Production of Pro-Inflammatory Cytokines by NHDC Metabolite in Macrophages

**Figure 1:**
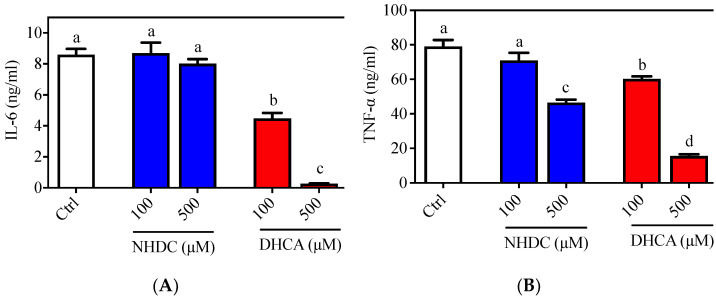
Pro-inflammatory cytokine secretion of LPS stimulated RAW 264.7 macrophages. Typical pro-inflammatory cytokines i.e., (**A**) IL-6 and (**B**) TNF-α were quantified by ELISA. Data are presented as means ± SEMs (*n* = 3). ^a–d^ Statistically significant values (*p* < 0.05) are displayed with a different letter. (NHDC, neohesperidin dihydrochalcone; DHCA, dihydrocaffeic acid).

The inflammatory responses of macrophages were evaluated in the RAW 264.7 cell line, which is a well-established cellular model for etiological studies [26,27]. Following LPS stimulation, an agonist of the toll-like receptor (TLR) 4 on the macrophage membranes, RAW 264.7 cells produced both IL-6 (8.596 ± 0.377 ng/mL) and TNF-α (79.099 ± 3.714 ng/mL) in the culture media (Figure 1, white bars). Treatment of cells with NHDC, in the presence of LPS, did not affect IL-6 secretion at concentrations of 100 or 500 μM (8.698 ± 0.674 ng/mL and 8.016 ± 0.298 ng/mL, respectively) (Figure 1A, blue bars). However, treatment with DHCA, the metabolite of NHDC, led to significantly reduced IL-6 production (4.484 ± 0.350 ng/mL for 100 μM and 0.266 ± 0.017 ng/mL for 500 μM) in a dose-dependent manner (Figure 1A, red bars). There was a comparable reduction of TNF-α secretion, as shown in Figure 1B. Briefly, treatment with a high concentration of NHDC (500 μM) led to a significant reduction in TNF-α (46.559 ± 1.762 ng/mL) compared to that in the LPS treated control (Figure 1B, blue bars), where treatment with 100 μM NHDC did not influence the TNF-α level (71.002 ± 3.714 ng/mL) compared to that in the LPS treated control. In contrast, DHCA treatment exhibited a dose-dependent reduction of TNF-α at concentrations of 100 μM (60.294 ± 1.451 ng/mL) and 500 μM (15.624 ± 0.967 ng/mL). These results imply that NHDC is a promising reagent for the regulation of the inflammatory responses in macrophages. The metabolism of NHDC to DHCA is critical for these responses.

In this regard, a previous study demonstrated that oral administration of NHDC prevented paraquat-induced hepatic inflammation in mice [19]. In addition, NHDC-fed mice demonstrated suppressed inflammatory responses through suppression of nuclear factor (NF)-κB activation, which is tightly controlled by TLR4 reception and the subsequent myeloid differentiation primary response (MyD)88 pathway [28]. However, it is unclear if NHDC directly affects the inflammatory cues in vivo. In the current cellular model system, NHDC only has a marginal effect on the suppression of inflammatory responses. Rather, its metabolite DHCA has potent anti-inflammatory properties.

### 3.2. Recovery of Mitochondrial Respiration by NHDC and DHCA in Macrophages

**Figure 2:**
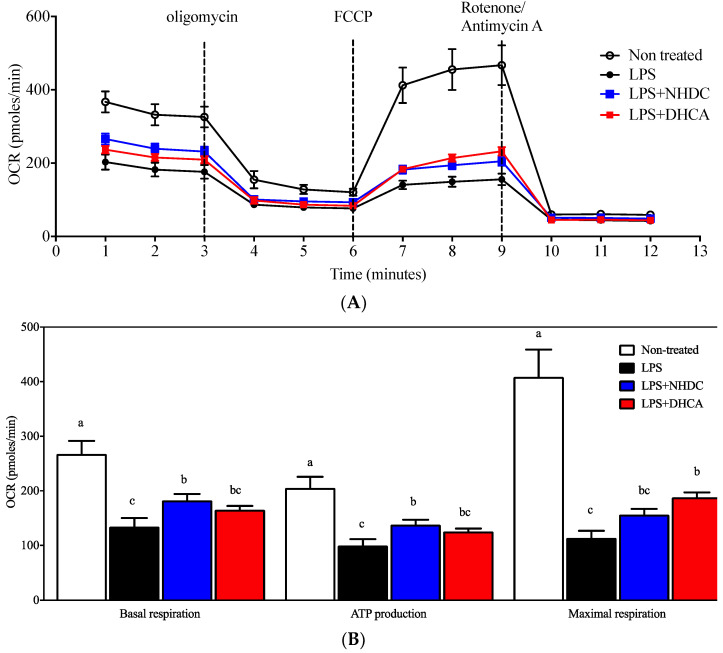
Mitochondrial respiration measurement by OCR. Mitochondrial respiration of RAW 264.7 macrophages was determined using a Seahorse extracellular flux analyzer by OCR (OCR, oxygen consumption rate). (**A**) Real-time OCR value of cells with NHDC or DHCA, with several drugs to regulate mitochondrial respiration. (**B**) Several respiration indices calculated by OCR, including basal respiration, maximal respiration, and ATP production. ^a–c^ Statistically significant values (*p* < 0.05) are displayed with different letters.

Previous reports suggest that macrophages are polarized into inflammatory M1 or regulatory M2 cells depending on the micromilieu of activating cues [29]. In this regard, the dichotomy of M1 vs. M2 cells is modulated by dietary intervention, in part by affecting cellular energy metabolism (i.e., glycolysis vs. mitochondrial respiration) [8]. In an effort to assess detailed regulation in this study, mitochondrial respiration was determined by the oxygen consumption rate (OCR) using the Seahorse extracellular flux analyzer. Non-stimulated control RAW 264.7 cells demonstrated an ordinary respiration pattern that is comparable to that of cellular metabolic capacity-determining drugs such as oligomycin, FCCP, and rotenone/antimycin A (Figure 2A, white circles) [30]. In contrast, an LPS-induced inflammatory response reduced the OCR of RAW264.7 cells (Figure 2A, black circles), for which NHDC (blue squares) or DHCA (red squares) treatment significantly reverted to the inclination of non-LPS treated cells. The dissected quantitative analyses were performed as described in the Materials and Methods section (Figure 2B). In detail, the assessment of OCR without any drug treatment counts for both cellular respiratory and non-respiratory oxygen consumption. Treatment with rotenone/antimycin A shuts down all of the cellular oxidative phosphorylation machineries. Therefore, basal respiration can be determined by subtracting the final OCR from the initial OCR and was significantly reduced by the addition of LPS (134.34 ± 15.81 pmoles/min) as compared to the non-treated control (267.52 ± 24.07 pmoles/min). Interestingly, the addition of NHDC significantly (*p* < 0.05) up-regulated OCR at 182.65 ± 11.73 pmoles/min as compared to LPS control. However, DHCA exhibited a marginal tendency (165.26 ± 7.15 pmoles/min) towards the non-stimulated control group. Following the treatment with oligomycin, which inhibits ATP synthase by blocking its proton channel, the cellular demand for oxygen drops over time (3–6 min, Figure 2A). This decrease can be calculated as the cellular activity of ATP production. There were comparable trends among the treatment groups in the OCR for ATP production compared to those with basal respiration (non-stimulated control 205.38 ± 20.24; LPS 99.72 ± 11.75; LPS + NHDC 138.12 ± 8.91; LPS + DHCS 125.63 ± 5.21 pmoles/min). The addition of FCCP to the cells maximizes the cellular demand for oxygen by uncoupling the oxidative phosphorylation machinery as seen at 6-9 min in Figure 2A. We calculated the difference in the maximal OCR after FCCP and OCR after rotenone/antimycin A treatment to assess the cellular capacity for maximal respiration. The OCR for maximal respiration decreased after LPS treatment (113.82 ± 12.95 pmoles/min) from the non-treated control (408.67 ± 50.30 pmoles/min). Interestingly, the addition of NHDC exhibited an increase of OCR (156.31 ± 10.62 pmoles/min) in the maximal respiration. DHCA treatment significantly up-regulated the maximal respiration at 188.32 ± 8.81 pmoles/min compared to that of the LPS treated control. Taken together, the analysis of cellular energy metabolism using the OCR assessment in dissected cellular responses revealed that both NHDC and DHCA had statistically significant effects on reversing the LPS-induced OCR decrease toward that of untreated cells.

### 3.3. Suppressed Lipid Accumulation in 3T3-L1 Adipocytes by DHCA

**Figure 3:**
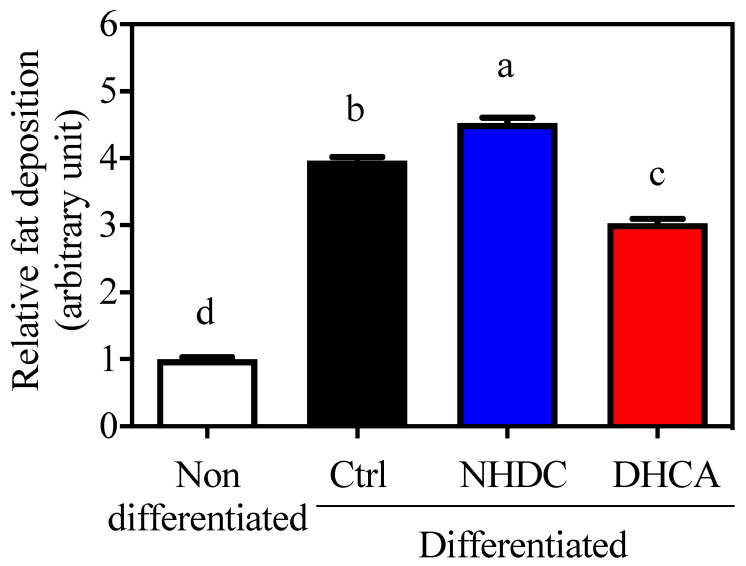
Suppressed lipid accumulation on 3T3-L1 by DHCA Relative Oil Red O absorbance of 3T3-L1 adipocytes with 500 μM of NHDC or DHCA treatment. 3T3-L1 preadipocytes differentiated into adipocytes with NHDC or DHCA. After 10 days from differentiation, adipocytes were stained with Oil Red O. Fat deposition was quantified by absorbance at 490 nm. The relative values are calculated by setting the Abs of the non-differentiated group as 1. The results were reported as means ± SEMs (*n* = 4). ^a–d^ Statistical significance is shown with different letters (*p* < 0.05).

Adipocytes are closely relevant to chronic inflammatory states because they secrete various adipokines that affect immune cell metabolism. In this regard, 3T3-L1 cells, a model for adipocytes, are widely used for the assessment of fat deposition by staining lipid droplets with ORO. Cellular differentiation to adipocytes was confirmed by increased fat deposition (3.96 ± 0.06, Figure 3, black bar, arbitrary unit) as compared to non-differentiated cells (1.00 ± 0.03, white bar). The adipocyte differentiation in the presence of NHDC showed a significant increment in fat deposition at 4.53 ± 0.08 (blue bar). However, DHCA drastically inhibited fat deposition to 3.03 ± 0.06 (red bar). It is important to note that NHDC contains two units of sugars in its chemical structure [31]. Its cellular degradation may affect cellular lipid deposition. However, current data clearly demonstrate that DHCA, a metabolite of NHDC, aids in the reduction of adipocyte function.

### 3.4. Regulation of Obesity-Induced Low-Grade Chronic Inflammation by Dietary NHDC

**Figure 4:**
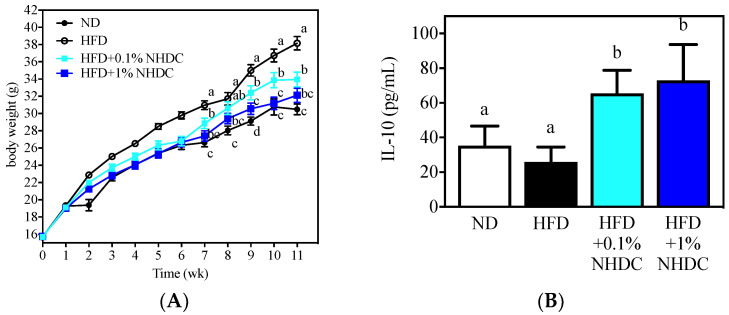
Body weight changes (**A**) and anti-inflammatory IL-10 secretion by M2-polarized BMDM of C57BL/6J male mice following 11 weeks of ad libitum dietary intervention (mean ± SEM, *n* = 10). ^a–c^ The different letters indicate statistical significance within the week (**A**) or the repeated experiment (**B**).

Following observations of the anti-inflammatory and anti-obesity properties of NHDC and its metabolite DHCA in vitro, the dietary effects were sought in an obese mouse model using HFD-fed C57BL/6J male mice. During the 11-week period of ad libitum feeding, the HFD-fed mice (Figure 4, white circles) gained significantly more body weight (starting at week 2) than did the ND-fed mice (black circles). In contrast, the body weight gain was significantly reduced by the NHDC-containing diet in a dose-dependent manner. In detail, 1% of NHDC (blue squares) in the HFD led to a similar body weight gain to that of the ND-fed mice throughout the dietary intervention. Mice with lower doses of NHDC (0.1% cyan squares), however, exhibited significantly (*p* < 0.05) more body weight gain after week 7 than did the ND-fed mice. It also should be noted that 0.1% NHDC exhibited significant suppression of high-fat-induced weight gain compared to that in the HFD-fed group. In order to clarify that the body change was not affected by the dietary intake, the daily consumption of diet per cage was weighed and divided by the number of mice in the cage, resulting in no difference among dietary intervention (ND 3.02 ± 0.68, HFD 2.99 ± 0.95, HFD + 0.1% NHDC 2.95 ± 0.62, and HFD + 1% NHDC 2.79 ± 0.55 g diet/day). These results clearly show that dietary NHDC suppresses body weight gain.

The bone marrow was collected from sacrificed mouse femurs and tibias and polarized to anti-inflammatory M2 macrophages according to previously reported protocols [32,33]. The culture media was harvested, and the IL-10 cytokine was quantified using ELISA. Interestingly, the high-fat diet did not affect IL-10 secretion (25.87 ± 8.68 pg/mL) compared to that in the ND-fed group (35.20 ± 11.41pg/mL). In contrast, the dietary NHDC at both low (0.1%) and high (1%) doses significantly increased IL-10 secretion at 65.46 ± 13.31 and 73.05 ± 20.56 pg/mL, respectively. These data support the in vitro observations that NHDC contributes to the suppression of lipid deposition and inflammatory responses in adipocytes and macrophages. These results also suggest that NHDC can be used as a therapeutic agent for chronic inflammatory responses that are induced by obesity.

## 4. Conclusions

NHDC is a sugar replacement product with high sweetness. Its chemical structure consists of polyphenolic compounds, which gives it a potentially beneficial health role. The current study evaluated the anti-inflammatory effects of NHDC and its biological metabolite DHCA. As observed in in vitro models, NHDC exhibited a marginal anti-inflammatory function, while DHCA had potent effects in a dose-dependent manner (Figure 1). Mechanistic studies using extracellular flux analysis have shown that both NHDC and DHCA reverted LPS-induced suppression of oxidative phosphorylation quantified as OCR (Figure 2A). Further experiments found that the cellular capacity of oxidative phosphorylation, which is a hallmark of anti-inflammatory macrophages [32], was rescued by both NHDC and DHCA (Figure 2B). These data support that NHDC and its biological metabolite DHCA modulate inflammatory responses, in part, by up-regulating cellular oxidative phosphorylation. DHCA, but not NHDC, down-regulated fat deposition in adipocytes in vitro. This finding indicates that the sugar moiety in NHDC may contribute to the lipid content following cellular uptake and metabolism (Figure 3). The in vitro results were also demonstrated in a mouse model in which HFD-induced obesity and low-grade chronic inflammation were established. Interestingly, dietary intake of NHDC ad libitum successfully suppressed body weight gain in a dose-dependent manner in the HFD-fed mice (Figure 4A). The production of the anti-inflammatory cytokine IL-10 was not affected in M2-polarized bone-marrow-derived macrophages from HFD-fed mice as compared to the ND control group. However, both NHDC and DHCA supplementation in HFD up-regulated the production of IL-10, suggesting the inflammatory status in obese status might be ameliorated. The data clearly indicate that NHDC and its metabolite DHCA possess anti-inflammatory properties both in vitro and in vivo. However, the molecular mechanisms involved in cellular signaling require additional investigation.

## Figures and Tables

**Table 1:** Experimental diet composition.

Composition (g/kg)				
Ingredients	ND	HFD	HFD + 0.1% NHDC	HFD + 1% NHDC
Casein	200	200	200	200
DL-methionine	3	3	3	3
Sucrose	500	370	370	370
Corn starch	150	111	111	111
Corn oil	50	30	30	30
Lard	-	170	170	170
Cellulose	50	50	49	40
Mineral mix S10001	35	35	35	35
Vitamin mix V1001	10	10	10	10
Choline bitartrate	2	2	2	2
NHDC	-	-	1	10

## References

[1] Marti A., Marcos A., Martinez J.A. (2001). Obesity and immune function relationships. Obes. Rev..

[2] Schmid-Schönbein G.W. (2006). Analysis of inflammation. Annu. Rev. Biomed. Eng..

[3] Dunster J.L. (2016). The macrophage and its role in inflammation and tissue repair: Mathematical and systems biology approaches. Wiley Interdiscip. Rev. Syst. Biol. Med..

[4] Sica A., Mantovani A. (2012). Macrophage plasticity and polarization: In vivo veritas. J. Clin. Investig..

[5] Liberti M.V., Locasale J.W. (2016). The Warburg effect: How does it benefit cancer cells?. Trends Biochem. Sci..

[6] Mills E.L., O’Neill L.A. (2016). Reprogramming mitochondrial metabolism in macrophages as an anti-inflammatory signal. Eur. J. Immunol..

[7] Choi Y., Ban I., Lee H., Baik M.-Y.M.-Y., Kim W. (2019). Puffing as a Novel Process to Enhance the Antioxidant and Anti-Inflammatory Properties of *Curcuma longa* L. (Turmeric). Antioxidants.

[8] Lee S., Yu S., Park H.J.H.J., Jung J., Go G.W.G.-W., Kim W. (2019). Rice bran oil ameliorates inflammatory responses by enhancing mitochondrial respiration in murine macrophages. PLoS ONE.

[9] Yu S., Go G.W., Kim W. (2019). Medium chain triglyceride (MCT) oil affects the immunophenotype via reprogramming of mitochondrial respiration in murine macrophages. Foods.

[10] Hotamisligil G.S. (2006). Inflammation and metabolic disorders. Nature.

[11] Weisberg S.P., McCann D., Desai M., Rosenbaum M., Leibel R.L., Ferrante A.W. (2003). Obesity is associated with macrophage accumulation in adipose tissue. J. Clin. Investig..

[12] Castoldi A., Naffah de Souza C., Câmara N.O.S., Moraes-Vieira P.M. (2016). The macrophage switch in obesity development. Front. Immunol..

[13] Coppack S.W. (2001). Pro-inflammatory cytokines and adipose tissue. Proc. Nutr. Soc..

[14] Frydman A., Weisshaus O., Huhman D.V., Sumner L.W., Bar-Peled M., Lewinsohn E., Fluhr R., Gressel J., Eyal Y. (2005). Metabolic Engineering of Plant Cells for Biotransformation of Hesperedin into Neohesperidin, a Substrate for Production of the Low-Calorie Sweetener and Flavor Enhancer NHDC. J. Agric. Food Chem..

[15] Belloir C., Neiers F., Briand L. (2017). Sweeteners and sweetness enhancers. Curr. Opin. Clin. Nutr. Metab. Care.

[16] Prinz P. (2019). The role of dietary sugars in health: Molecular composition or just calories?. Eur. J. Clin. Nutr..

[17] Waalkens-Berendsen D., Kuilman-Wahls M.E., Bär A. (2004). Embryotoxicity and teratogenicity study with neohesperidin dihydrochalcone in rats. Regul. Toxicol. Pharmacol..

[18] Lina B.A.R., Dreef-van der Meulen H.C., Leegwater D.C. (1990). Subchronic (13-week) oral toxicity of neohesperidin dihydrochalcone in rats. Food Chem. Toxicol..

[19] Shi Q., Song X., Fu J., Su C., Xia X., Song E., Song Y. (2015). Artificial sweetener neohesperidin dihydrochalcone showed antioxidative, anti-inflammatory and anti-apoptosis effects against paraquat-induced liver injury in mice. Int. Immunopharmacol..

[20] Suarez J., Herrera M.D., Marhuenda E. (1998). In vitro scavenger and antioxidant properties of hesperidin and neohesperidin dihydrochalcone. Phytomedicine.

[21] Garg A., Garg S., Zaneveld L.J.D., Singla A.K. (2001). Chemistry and pharmacology of the citrus bioflavonoid hesperidin. Phyther. Res..

[22] Wu J., Li J., Cai Y., Pan Y., Ye F., Zhang Y., Zhao Y., Yang S., Li X., Liang G. (2011). Evaluation and Discovery of Novel Synthetic Chalcone Derivatives as Anti-Inflammatory Agents. J. Med. Chem..

[23] Yadav V.R., Prasad S., Sung B., Aggarwal B.B. (2011). The role of chalcones in suppression of NF-κB-mediated inflammation and cancer. Int. Immunopharmacol..

[24] Braune A., Engst W., Blaut M. (2005). Degradation of Neohesperidin Dihydrochalcone by Human Intestinal Bacteria. J. Agric. Food Chem..

[25] Kwon Y., Yu S., Choi G.S., Kim J.H., Baik M., Su S.T., Kim W. (2019). Puffing of Rehmannia glutinosa enhances anti-oxidant capacity and down-regulates IL-6 production in RAW 264.7 cells. Food Sci. Biotechnol..

[26] Tanoue T., Nishitani Y., Kanazawa K., Hashimoto T., Mizuno M. (2008). In vitro model to estimate gut inflammation using co-cultured Caco-2 and RAW264.7 cells. Biochem. Biophys. Res. Commun..

[27] Lee J.Y., Yoo J.-M., Baek S.Y., Kim M.R. (2019). Anti-dermatitic effect of fermented ginseng extract including rich compound K through inhibiting activation of macrophage. Food Sci. Biotechnol..

[28] Xia X., Fu J., Song X., Shi Q., Su C., Song E., Song Y. (2015). Neohesperidin dihydrochalcone down-regulates MyD88-dependent and -independent signaling by inhibiting endotoxin-induced trafficking of TLR4 to lipid rafts. Free Radic. Biol. Med..

[29] Martinez F.O., Gordon S. (2014). The M1 and M2 paradigm of macrophage activation: Time for reassessment. F1000Prime Rep..

[30] Iuso A., Repp B., Biagosch C., Terrile C., Prokisch H. (2017). Assessing Mitochondrial Bioenergetics in Isolated Mitochondria from Various Mouse Tissues Using Seahorse XF96 Analyzer. Methods in Molecular Biology.

[31] Veitch N.C., Grayer R.J. (2011). Flavonoids and their glycosides, including anthocyanins. Nat. Prod. Rep..

[32] Feng J., Li L., Ou Z., Li Q., Gong B., Zhao Z., Qi W., Zhou T., Zhong J., Cai W. (2018). IL-25 stimulates M2 macrophage polarization and thereby promotes mitochondrial respiratory capacity and lipolysis in adipose tissues against obesity. Cell. Mol. Immunol..

[33] Lee S., Kim W. (2018). Effects of dietary rice bran oil on mitochondrial respiration in M2-induced bon marrow-derived macrophages. Food Eng. Prog..

